# The Foreign Body Giant Cell Cannot Resorb Bone, But Dissolves Hydroxyapatite Like Osteoclasts

**DOI:** 10.1371/journal.pone.0139564

**Published:** 2015-10-01

**Authors:** Bas ten Harkel, Ton Schoenmaker, Daisy I. Picavet, Noel L. Davison, Teun J. de Vries, Vincent Everts

**Affiliations:** 1 Department of Oral Cell Biology, Academic Centre for Dentistry Amsterdam (ACTA), MOVE Research Institute, University of Amsterdam and VU University Amsterdam, Amsterdam, The Netherlands; 2 Department of Periodontology, Academic Centre for Dentistry Amsterdam (ACTA), MOVE Research Institute, University of Amsterdam and VU University Amsterdam, Amsterdam, The Netherlands; 3 Department of Cell Biology and Histology, Center for Advanced Microscopy, Academic Medical Center, University of Amsterdam, Amsterdam, The Netherlands; 4 MIRA Institute for Biomedical Technology and Technical Medicine, University of Twente, Enschede, The Netherlands; 5 Xpand Biotechnology BV, Bilthoven, The Netherlands; University of Oulu, FINLAND

## Abstract

Foreign body multinucleated giant cells (FBGCs) and osteoclasts share several characteristics, like a common myeloid precursor cell, multinuclearity, expression of tartrate-resistant acid phosphatase (TRAcP) and dendritic cell-specific transmembrane protein (DC-STAMP). However, there is an important difference: osteoclasts form and reside in the vicinity of bone, while FBGCs form only under pathological conditions or at the surface of foreign materials, like medical implants. Despite similarities, an important distinction between these cell types is that osteoclasts can resorb bone, but it is unknown whether FBGCs are capable of such an activity. To investigate this, we differentiated FBGCs and osteoclasts in vitro from their common CD14^+^ monocyte precursor cells, using different sets of cytokines. Both cell types were cultured on bovine bone slices and analyzed for typical osteoclast features, such as bone resorption, presence of actin rings, formation of a ruffled border, and characteristic gene expression over time. Additionally, both cell types were cultured on a biomimetic hydroxyapatite coating to discriminate between bone resorption and mineral dissolution independent of organic matrix proteolysis. Both cell types differentiated into multinucleated cells on bone, but FBGCs were larger and had a higher number of nuclei compared to osteoclasts. FBGCs were not able to resorb bone, yet they were able to dissolve the mineral fraction of bone at the surface. Remarkably, FBGCs also expressed actin rings, podosome belts and sealing zones—cytoskeletal organization that is considered to be osteoclast-specific. However, they did not form a ruffled border. At the gene expression level, FBGCs and osteoclasts expressed similar levels of mRNAs that are associated with the dissolution of mineral (e.g., anion exchange protein 2 (AE2), carbonic anhydrase 2 (CAII), chloride channel 7 (CIC7), and vacuolar-type H^+^-ATPase (v-ATPase)), in contrast the matrix degrading enzyme cathepsin K, which was hardly expressed by FBGCs. Functionally, the latter cells were able to dissolve a biomimetic hydroxyapatite coating in vitro, which was blocked by inhibiting v-ATPase enzyme activity. These results show that FBGCs have the capacity to dissolve the mineral phase of bone, similar to osteoclasts. However, they are not able to digest the matrix fraction of bone, likely due to the lack of a ruffled border and cathepsin K.

## Introduction

Cell types with more than one nucleus are relatively rare in our body. Under physiological conditions three different cell types are recognized with more than one nucleus: (i) skeletal muscle cells, (ii) the syncytiotrophoblast of the mature placenta, and (iii) the osteoclast. Myoblasts [1] fuse to form skeletal muscle, trophoblasts of the placenta fuse to form the syncytiotrophoblasts [2], and monocytes fuse to generate osteoclasts [3].

Multinuclearity is considered to be beneficial for the functioning of these different cell types. It allows rapid coordination of muscle fiber contraction along the whole length of the muscle fiber, protects the placenta from invading immune cells which can trigger an immune response [2], and it enables the osteoclast to be more efficient in resorbing mineralized tissues [4].

Under certain pathological conditions a different type of multinucleated cell can be formed: the FBGC. This cell type originates, like the osteoclast, from fusion of monocytes/macrophages [5]. The formation of FBGCs occurs at the surface of foreign materials, like implants. Such biomedical devices or tissue-engineered constructs are used in a wide variety of applications like vascular stents, dental restorations and artificial hips. Whether formation of FBGCs occurs depends on the material used as well as its shape, size, surface chemistry, roughness, morphology and design [6–8]

Different hypotheses attempt to explain what triggers FBGC formation. One theory suggests that when macrophages encounter a particle too big to be phagocytosed by a single cell, they fuse to form an FBGC in an attempt to engulf it—so called “frustrated phagocytosis”. Another theory is that fusion could be an escape mechanism to avoid apoptosis. When macrophages cannot attach to a biomaterial they become apoptotic; to prevent apoptosis they fuse and become FBGCs [9]. A third hypothesis is that they protect surrounding tissue from a foreign material by forming a barrier at the tissue-material interface [10]. Moreover, the exact function of FBGCs is also unclear.

To understand more about the function of FBGCs, one could compare them with osteoclasts, which share many similarities [11–15]. In addition to being multinucleated, both cell types arise from fusion of monocytes and express high levels of TRAcP. Recently some fusion proteins have been discovered in both cell types such as DC-STAMP [16], and osteoclast stimulatory transmembrane protein (OC-STAMP) [11]. There appears to be, however, at least one essential difference between the two cell types: their ability to resorb bone.

Osteoclasts are unique in their capacity to digest the mineralized tissue, whereas FBGC are not known to share this ability. However, FBGCs have been implicated with bone loss around oral implants [17–19], suggesting that FBGCs may also be able to resorb bone. Yet, no direct evidence has been presented to demonstrate this, nor has the function of FBGCs been compared to that of osteoclasts on bone.

In order to investigate the possibility that FBGCs may be able to resorb bone like their multinucleated cousins, the osteoclasts, we differentiated both cell types from human CD14^+^ monocyte precursors using different sets of cytokines in vitro. Specifically, it has been shown that interleukin-4 (IL-4) and interleukin-13 (IL-13) can induce the formation of FBGCs [20,21], whereas receptor activator of nuclear factor kappa-B ligand (RANK-L), in conjunction with macrophage colony-stimulating factor (M-CSF), is a inducer of osteoclasts. As mononuclear controls, monocytes were cultured solely in the presence of M-CSF (generating macrophages) or without any cytokines. Cell types were cultured on bovine bone slices as well as on biomimetic hydroxyapatite coatings, and analyzed for bone resorption, mineral dissolution, actin ring organization, ruffled border formation, and gene expression over time.

## Materials and Methods

### CD14^+^ cell isolation

Buffy coats containing peripheral mononuclear cells (PBMCs) were purchased from Sanquin (Amsterdam, The Netherlands). The buffy coats were mixed with a buffer consisting of sodium citrate-2 (1.55 M), citric acid-1 (0.11 M) and phosphate-buffered saline (PBS). Ficoll was used to separate mononuclear cells from red blood cells. The ficoll gradient with the PBMCs and red blood cells were centrifuged for 30 min (800xg, no brake). After centrifugation the interphase containing the PBMCs was collected. The PBMCs were centrifuged for 10 min at 400xg (with brake). Supernatant was decanted and cells were resuspended in the citrate buffer. This washing step was repeated 3 times. After the washing steps 20 ml of buffer, consisting of PBS, 0.5% bovine serum albumin (BSA), and 2 mM Ethylene diaminetetra-acetic acid (EDTA) was added and used for all consecutive washing steps. The cells were counted with a Muse Cell Analyzer (Merck Millipore, Billerica, MA).

The cell number was determined and the cells were centrifuged for 10 min at 400xg (with brake). The supernatant was aspirated and the PBMCs were resuspended in 80 μl of buffer per 10^7^ cells. A manual magnetic assisted cell sorter (MACS) and iron conjugated CD14 antibodies (Miltenyi Biotec, Bergisch Gladbach, Germany) were used to isolate CD14^+^ cells. Per 10^7^ cells, 20 μl of CD14 microbeads was added and incubated for 15 min in the refrigerator (2–8°C) according to the manufacturer’s instructions (Miltenyi Biotec). After 15 min of labeling with anti-CD14 beads, buffer was added and the cells were pelleted for 10 min at 400xg. Supernatant was aspirated and the PBMCs were resuspended in 500 μl of buffer. A magnetic column was placed in the magnetic field of the MACS separator. The column was rinsed with 3 ml buffer. The PBMC suspension was applied to the column. The column was rinsed 3x3 ml, allowing non-labeled cells to come off the column. Next, the column was removed from the magnet and 5 ml buffer was added to collect the CD14^+^ cells. The buffer was pressed over the column with a plunger and hence the cells were collected in a tube. The cell density was determined and after collection of the appropriate number of CD14^+^ cells, the cells were centrifuged for 10 min at 400xg (with brake). Supernatant was aspirated and culture medium was added. The culture medium consisted of alpha minimum essential medium (αMEM; Life Technologies, Carlsbad, CA), 10% fetal calf serum (FCS; HyClone, Logan, UT) and 1% penicillin/streptomycin/fungisone (PSF; Sigma-Aldrich, Saint Louis, MO).

To analyze the purity of the isolation, a fraction of the cells was labeled with a FITC conjugated mouse anti-CD14 antibody (Miltenyi Biotec). Subsequent flowcytometric analysis revealed that the average purity of CD14^+^ cells of 5 independent experiments was 83% ± 4% (mean ± SD). FITC-labeled mouse IgG2a isotype antibody (Miltenyi Biotec) served as labeling control.

### Cell culture

The CD14^+^ cells were seeded on bovine cortical bone in 96-well plates. Bone slices were 0.5 mm thick. The cell concentration was 1.5 x 10^5^ cells per 0.32 cm^2^ (surface of one well). The cells were cultured for 3 days with 25 ng/ml human recombinant M-CSF (R&D Systems, MI). After 3 days the concentration of M-CSF was reduced to 10 ng/ml for both FBGC and osteoclast cultures until the end of the culture period. For the generation of FBGCs 5 ng/ml human recombinant IL-4 and 5 ng/ml human recombinant IL-13 were added to the cultures. For the generation of osteoclasts 2 ng/ml mouse recombinant RANK-L (R&D systems) was added. The cell density and the cytokine concentration for the generation of FBGCs and osteoclasts were carefully titrated. Deviations from the optimal cell density and cytokine concentration lead to apoptosis or no formation of multinucleated cells. As controls we used two cultures, one with only M-CSF and one without cytokines. The M-CSF culture received the same pre-treatment as the other cultures and after 3 days the concentration was reduced to 10 ng/ml. The culture medium was refreshed every 3–4 days. The total culture time lasted for 18 and 25 days.

### Preparation of hydroxyapatite coatings on 96-well plates

The coating process consisted of two steps:

*Step 1*: *pre-calcification*: 96-well plates were pre-coated by pipetting a 3 times supersaturated Simulated Body Fluid (SBF) solution into the wells (250 μl/well). In order to prepare SBF 3x solution, the following three solutions were made: (1) Buffer solution by dissolving 24.2 g Tris base and 164 ml 1M HCl in MilliQ water to a total volume of 4L (final pH = 7.4); (2) calcium solution by dissolving 24 g NaCl, 0.9 g MgCl_2_ · 6H_2_O and 1.2 g CaCl_2_ · 2H_2_O in 500 ml of buffer solution (#1), and (3) phosphate solution by dissolving 0.6 g NaHCO_3_ and 1.2 g Na_2_HPO_4_ · H_2_O in 500 ml buffer solution (#1). SBF was prepared by mixing the two latter solutions. SBF was refreshed every day during the 3 day incubation period. At the end of day 3, the plates were thoroughly washed (5x) with MilliQ water.
*Step 2*: *crystal growth*: Pre-coated 96-well plates were treated with a calcium phosphate supersaturated solution (CPS) at physiological pH of 7.4 (250 μl/well) to deposit a crystallized layer onto the amorphous calcium phosphate layer formed in step 1. CPS was prepared by dissolving 8.0 g NaCl, 0.59 g CaCl_2_ · 2H_2_O, 0.36 g Na_2_HPO_4_ · H_2_O and 6.05 Tris in 800 ml of MilliQ water, adjusting the pH of the solution to 7.4 using 1M HCl. We added 200 ml MilliQ water to the solution to reach 1000 ml. The CPS solution was added to a 96 well plate (250 μl per well). The coatings resemble to that of the hydroxyapatite in bone [22]. We seeded the cells in the same way as the cells cultured on bone and analyzed the dissolution of the hydroxyapatite coating by using ImageJ. Three control groups were used, CD14^+^ cells that were cultured without cytokines, CD14^+^ cells with M-CSF only, and fibroblasts (to check if another cell type would affect the hydroxyapatite coating).

### Histological staining

After 18 and 25 days the osteoclasts and FBGCs were fixed for 10 min with 4% PBS buffered formaldehyde. The cells were stained for TRAcP activity (acid phosphatase leukocyte kit; Sigma-Aldrich) and with 4',6-diamidino-2-fenylindool (DAPI). The number of multinucleated cells on the bone slices were counted and related to the surface area of the bone using ImageJ. The multinucleated cells were categorized into one of the following three groups: 3 to 5 nuclei per cell, 6 to 10 nuclei per cell and more than 10 nuclei per cell.

To visualize bone resorption, bone slices were stained with coomassie brilliant blue (CBB; GE Healthcare life sciences, NJ). The bone slices were washed with milliQ water and sonicated for 30 min in 10% NH_3_OH on ice. After sonification the bone slices were washed with milliQ water and transferred to a new well. The bone slices were washed in water saturated alum and incubated with water saturated alum for 10 min. After incubation the bone slices were washed twice with milliQ. Both sides of the bone chip were dried between filter paper and rinsed with CBB (10 ml) and subsequently dried between filter paper. The blue resorption pits were analyzed using ImageJ.

### Immunohistochemistry

After fixation of the cells on bone with 4% formaldehyde for 10 min, the slices were washed with PBS and incubated with 20% normal goat serum, then incubated for 1 hour at room temperature with mouse anti-human monoclonal anti-CD44 (Sigma-Aldrich) to stain the plasma membrane (1:100 dilution). Bone slices were washed 3 times for 5 min in PBS and stained with goat anti-mouse Alexa 647-conjugated secondary antibody (1:400) (Life Technologies). After washing (3x5 min PBS) the slices were stained with Alexa 488-conjugated phalloidin targeting F-actin (1:400) (Life Technologies), and Hoechst 33342 dye (1:500) (Sigma-Aldrich) to stain the nuclei [23].

Plasma membrane, actin rings and nuclei were visualized using fluorescent laser scanning confocal microscopy (Leica SP8-SMD; Leica DMI6000 microscope; Leica LAS AF software, Darmstadt, Germany). Overlaid image stacks were generated by scanning from the apical to the basal surface of the cell.

3,6-bis[dimethylamine]acridine (acridine orange) was used to visualize the compartments with a low pH. Acridine orange at 10 μg/mL was added 10 min prior to finishing the culture [24]. The dye was washed away and micrographs were taken using a Leica DM IL.

Concanamycin A (ConcA) (Sigma-Aldrich) was used to block v-ATPase activity[25]. In a preliminary series of experiments we assessed the concentration of ConcA that did not interfere with cell viability. This concentration was 25 ng/ml. ConcA was added to the cultures on the biomimetic hydroxyapatite coating at day 6 of the culture and at each refreshing step. Extra control groups were included (cells on bone slices and on plastic; because cells on the biomimetic hydroxyapatite coating are difficult to observe when they do not dissolve the coating) to which ConcA was added to confirm that the cells proliferated and differentiated into multinucleated cells.

### Transmission electron microscopy (TEM)

After culturing CD14^+^ cells under FBGC and osteoclast differentiation conditions on bone slices for 18 and 25 days, the slices were fixed overnight in 1% glutaraldehyde plus 4% formaldehyde in 0.1 M sodium cacodylate buffer. After decalcification in an EDTA solution (1.9% glutaraldehyde, 0.15 M EDTA in 0.06 M sodium cacodylate buffer) the bone slices were postfixed with a solution of 1% OsO4 in water. Subsequently, the specimens were dehydrated in an ethanol series and embedded in epoxy resin (LX112).

For light microscopic analysis 1 μm sections were made and stained with Richardson’s staining solution. For electron microscopic analysis ultrathin sections of the bone slices were collected on formvar-coated grids and counterstained with uranyl acetate and lead citrate. Images were acquired using a FEI Technai 12 transmission electron microscope.

### Quantitative PCR (qPCR)

Total RNA was isolated using a commercial spin-column kit (Qiagen, Hilden, Germany) following the manufacturer’s instructions (Qiagen). Bone slices containing cells were transferred to new wells containing RLT lysis buffer with 1% β-mercaptoethanol. After column purification of total RNA from the cell lysate, RNA concentration was measured using a Synergy spectrophotometer. Reverse transcription of RNA was performed using the MBI Fermentas cDNA synthesis kit (Fermentas, Lithuania), using both the Oligo(dT)18 and the D(N)6 primers.

Quantitative PCR (qPCR) was performed on an ABI PRISM 7000 Sequence Detection System. The PCR reactions were performed with 30 ng cDNA in a total volume of 15 μL containing SYBR GreenER qPCR SuperMix, consisting of SYBR Green 1 Dye, AmpliTaq Gold DNA polymerase, dNTPs, passive reference and buffer (Life Technologies), and 300 nM of primer. For gene targets, the protocol consisted of an activation step (10 min, 94°C) and 40 cycles of two-step PCR (95°C for 15 sec, 60°C for 1 min). TATAb housekeeping gene was analyzed using a three-step protocol (95°C for 10 sec, 55°C for 30 sec, 72°C for 30 sec). All genes were subjected to melt curve analysis to test for unspecific PCR products. Expression of gene targets was normalized by endogenous expression of two housekeeping genes (TATAb, GAPDH) following the comparative C_T_ method[26] and presented as fold expression (2^-ΔCT^). The housekeeping genes were shown to be stable using GeNorm software (v. 3) and their geometric mean expression was calculated for numerical normalization of gene targets. QPCR Primers were designed using Primer Express 2.0 software (Life Technologies) (Table 1), spanning at least 1 intron to avoid amplification of genomic DNA. QPCR analysis was conducted using 5 independent donors and 2–3 culture replicates per donor. Averages of these replicates were used to obtain one sample read-out.

**Table 1:** qPCR primer sequences.

Gene Target	Sequence (5’- 3’)	Product size (bp)	Accesion ID
GAPDH	atggggaaggtgaaggtcg	68	ENSG 00000149397
	taaaagcagccctggtgacc		
TATAb	ggtctgggaaaatggtgtgc	100	ENSG00000112592
	gctggaaaacccaacttctg		
DC-STAMP	attttctcagtgagcaagcagtttc	101	ENSG00000164935
	Agaatcatggataatatcttgagttcctt		
CD36	gtgatgatgaacagcagcaaca	100	ENSG00000135218
	tcctcagcgtcctgggttac		
ITGB3	Aggctggcaggcattgtc	100	ENSG00000259207
	agccccaaagagggataatcc		
c-SRC	gcactacaagatccgcaagct	106	ENSG00000197122
	catcggcgtgtttggagtagta		
COX IV	cctgtctgccagccagaag	101	ENSG00000131143
	tccttgaacttaatgcgatacaactc		
AE2	ttgtgggcctctccatagttatc	103	ENSG00000164889
	gatcccgttaagggaggtgact		
CAII	tggactggccgttctaggtatt	100	ENSG00000104267
	tcttgccctttgttttaatggaa		
CLC7	aaggtttgactcggagaaaatgg	100	ENSG00000103249
	aggcattgaacactgctccaa		
v-ATPase	gctgccaaccacttgagctt	114	ENSG00000110719
	caaagtgcacgtggttgaaga		
CR	gcataccaaggagaaggtccatat	78	ENSG00000004948
	atactccagccggtgtgtcat		
Cath K	ccatatgtgggacaggaagagagtt	149	ENSG00000143387
	tgcatcaatggccacagaga		
NFATc1	agcagagcacggacagctatc	143	ENSG00000131196
	ggtcagttttcgcttccatctc		

### Statistics

Statistical analyses were performed with paired sample T-test using SPSS. P values < 0.05 were considered significant.

## Results

### TRAcP activity and multinuclearity

**Fig 1:**
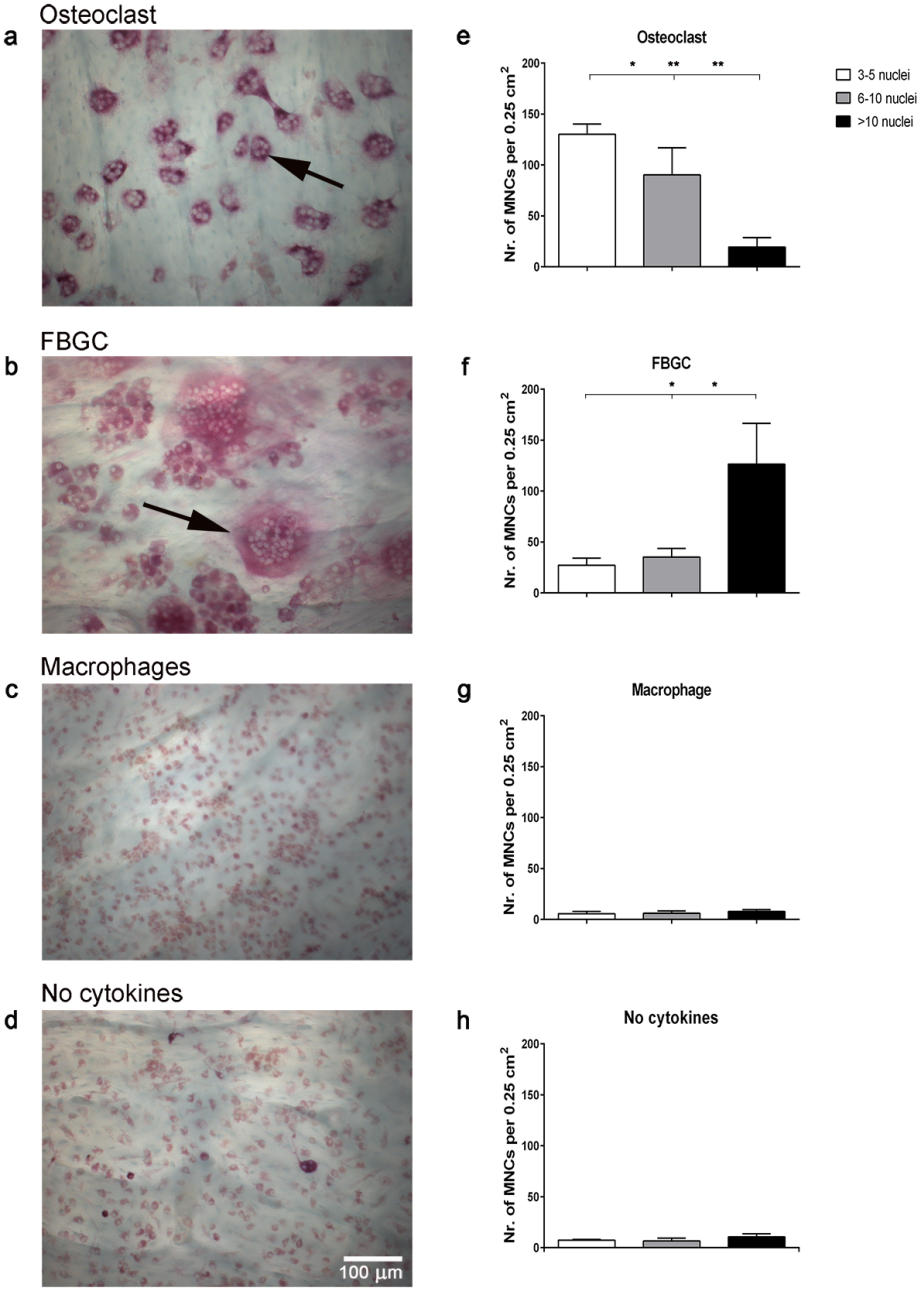
TRAcP activity of osteoclasts, FBGCs, and macrophages cultured on bone. Human CD14^+^ monocytes were cultured on bone slices for 25 days with M-CSF and RANK-L (osteoclasts); M-CSF, IL-4, and IL-13 (FBGCs); M-CSF (macrophages) and without cytokines (control). Osteoclasts were TRAcP-positive (**a**; black arrow) and most contained < 10 nuclei (**e**). FBGCs were larger with > 10 nuclei (**f**), and stained less intensely for TRAcP (**b**; black arrow). Macrophages were generally mononuclear and stained weakly for TRAcP (**c, g**), similar as CD14^+^ cells that were cultured without cytokines (**d, h**). Bar plots represent the mean ± S.D. of multinucleated cells (MNCs) per 0.25 cm^2^ bone surface, from 5 independent donors. Scale bar = 100 μm. Red asterisk = bone. *p<0.05, **p<0.01.

CD14^+^ cells fused to form multinucleated cells after culture for 18 and 25 days with cytokines that induced FBGCs (M-CSF, IL-4, and IL-13) as well as those that induced osteoclasts (M-CSF, and RANK-L). Fusion was hardly noticed in control cell cultures (i.e., CD14^+^ cells ± M-CSF). FBGCs were considerably larger and contained more nuclei per cell than osteoclasts (Fig 1e and 1f; number of cells with more than 10 nuclei: FBGCs: 127 ± 69 SD; osteoclasts: 19 ± 7 SD, p<0.01). The cells in the control cultures (± M-CSF) were much smaller.

All cell types stained positive for TRAcP activity, but staining intensity varied per cell type. Specifically, TRAcP staining was less intense for FBGCs compared to osteoclasts (Fig 1a), but more intense than that of the mononuclear cells (Fig 1c and 1d). For all cell types, TRAcP staining was distributed throughout the cytoplasm rather than localized in specific areas of the cells (Fig 1a-1d).

### Bone resorption by osteoclasts but not by FBGCs

**Fig 2:**
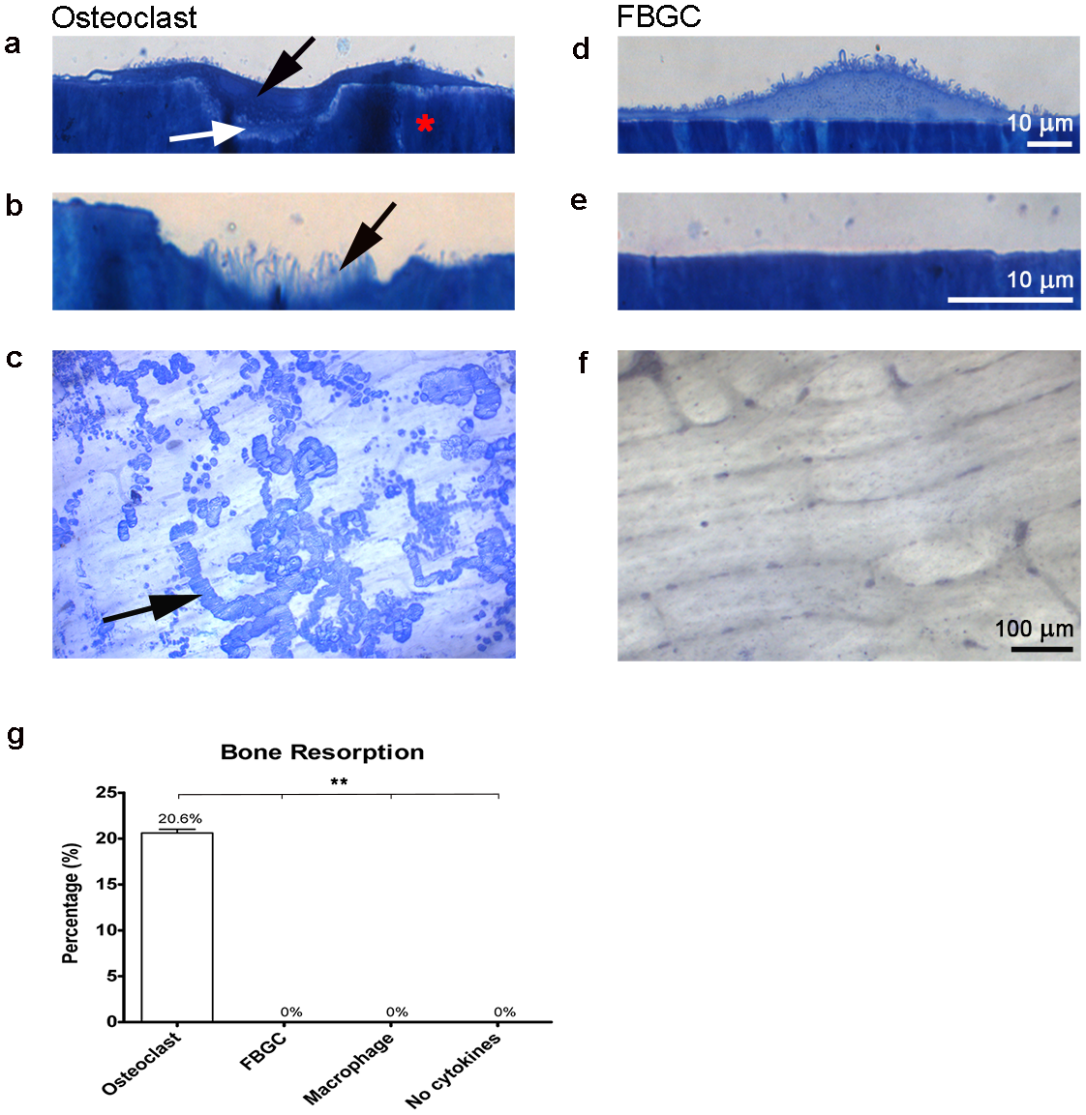
Bone resorption by osteoclasts and FBGCs. After 25 days, cells were stained with Richardson’s staining solution (**a-e**) and resorption pits were visualized (**c-f**) and quantified (g) using coomassie brilliant blue (CBB). Osteoclasts created resorption pits (Howship’s lacunae) (**a**; black arrow) and formed a ruffled border (white arrow). No resorption pits nor ruffled borders were visible in the FBGC cultures (**d, e**). In the resorption pits, collagen fibrils were visible (**b**; black arrow). Numerous resorption pits were seen in the osteoclast culture (**c**; black arrow), but no signs of resorption were apparent in the FBGC cultures. Osteoclasts resorbed more than 20% of the bone surface (**g**). The percent bone resorption graph represent the mean area ± S.D. per 0.25 cm^2^ bone surface. Scale bar is 10 μm for panels **a, b, d, e**. Scale bar is 100 μm for panels **c** and **f**. Red asterisk = bone. *p<0.05, **p<0.01.

Coomassie brilliant blue staining showed that osteoclasts formed numerous resorption pits on bone slices (Fig 2a-2c), covering ~20% of the bone surface (Fig 2g). In contrast, no resorption pits were detected in the FBGC cultures (Fig 2d-2f), although cells were present in high numbers (Fig 1). Similarly, no resorption pits were detected in the control mononuclear cultures.

To investigate whether bone resorption by FBGCs could be induced by RANK-L, this cytokine was added to FBGC cultures at day 21 and day 25 and incubated for 6 additional days with M-CSF, but without IL-4 and IL-13. These relatively late time points were chosen to ensure that the FBGC were fully differentiated. Still, no resorption was detected, indicating that mature FBGCs cannot resorb bone even in the presence of RANK-L.

### FBGCs show actin rings similar to osteoclasts

**Fig 3:**
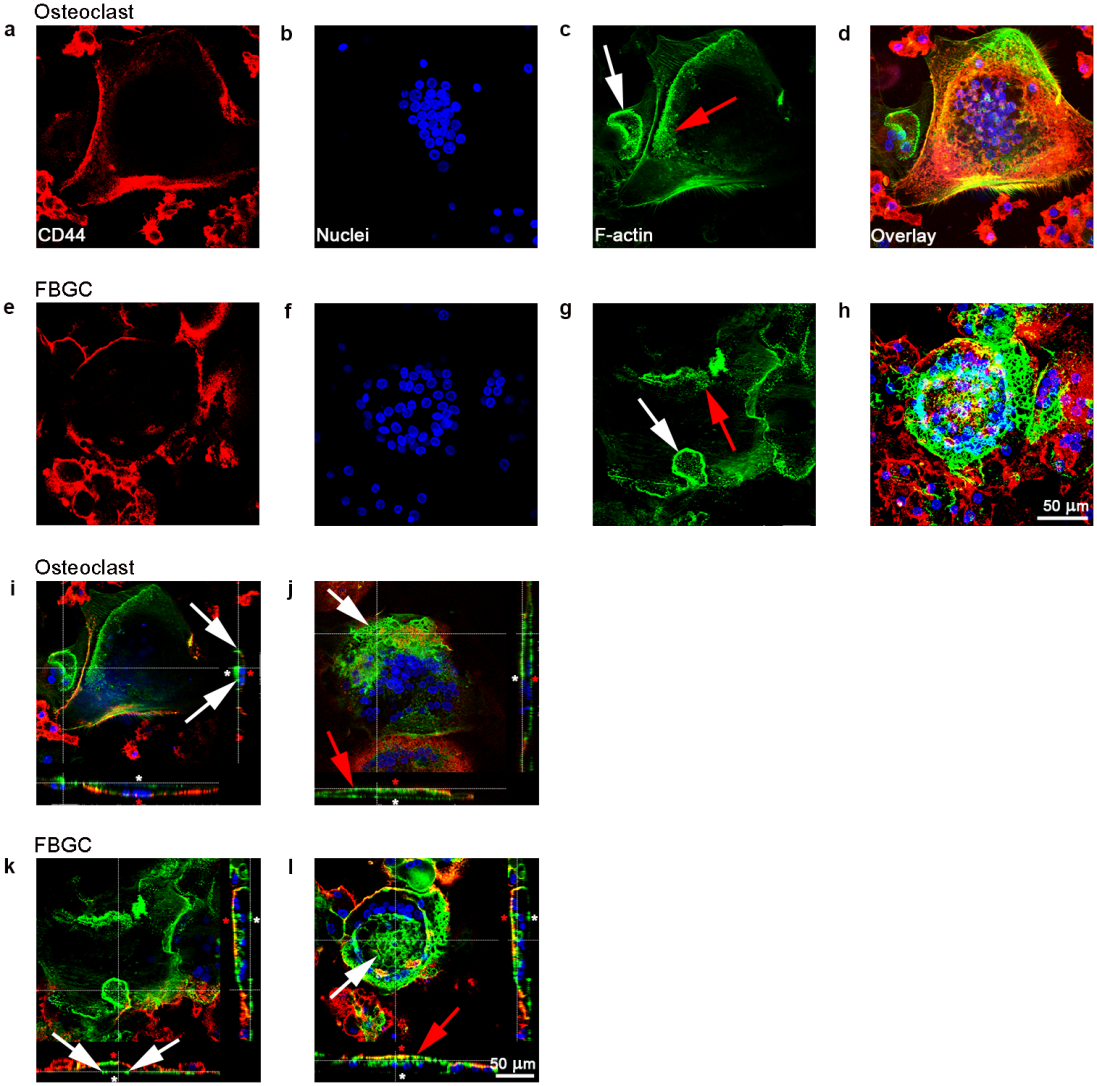
Confocal microscopy of osteoclasts and FBGCs. Plasma membrane (red: CD44 antibody), nuclei (blue: Hoechst nuclei staining), and actin rings (green: phalloidin staining of F-actin) were fluorescently labeled after 25 days culture on bone. Both cell types contained numerous nuclei (**a, b, e, f,**), actin rings (**c, g**; white arrow,), and podosome belts (**c, g**; red arrow). Sagittal views of both cell types composed from the apical side (white asterisk) showed actin structures resembling sealing zones (**i, k**; white arrows). Sagittal views composed from the basolateral side (red asterisk) of the cells showed round structures composed of actin (**j, I**; white arrow, red arrow). Scale bar = 50 μm.

Organization of the cell membrane (Fig 3a and 3e), nuclei (Fig 3b and 3f) and actin (Fig 3c and 3h) was analyzed using confocal microscopy. Osteoclasts formed numerous actin rings (Fig 3c). Mostly they were round of shape and had a diameter of approximately 15–25 μm. The actin rings formed near the cell membrane (Fig 3c). Interestingly, FBGCs also formed actin rings (Fig 3g). They had the same shape and diameter as those found in the osteoclasts. Podosomes—punctate actin condensations—were observed in both cell types (Fig 3c and 3g) and were located in the actin rings or near the plasma membrane. Cross-sectional image stacks showed that both cell types were closely associated with the bone surface (Fig 3i and 3k).

Another phenomenon seen in both cell types was a large actin-containing structure composed of different compartments on the baso-lateral side of the cell (Fig 3j and 3l). This actin structure started in the center of the cell and formed a kind of roof-like structure at the top or lateral part of the cell. This was not seen in mononuclear cells, therefore we assume that this structure provides extra cytoskeletal support because of the large size of the multinucleated cells.

### FBGCs do not form a ruffled border

**Fig 4:**
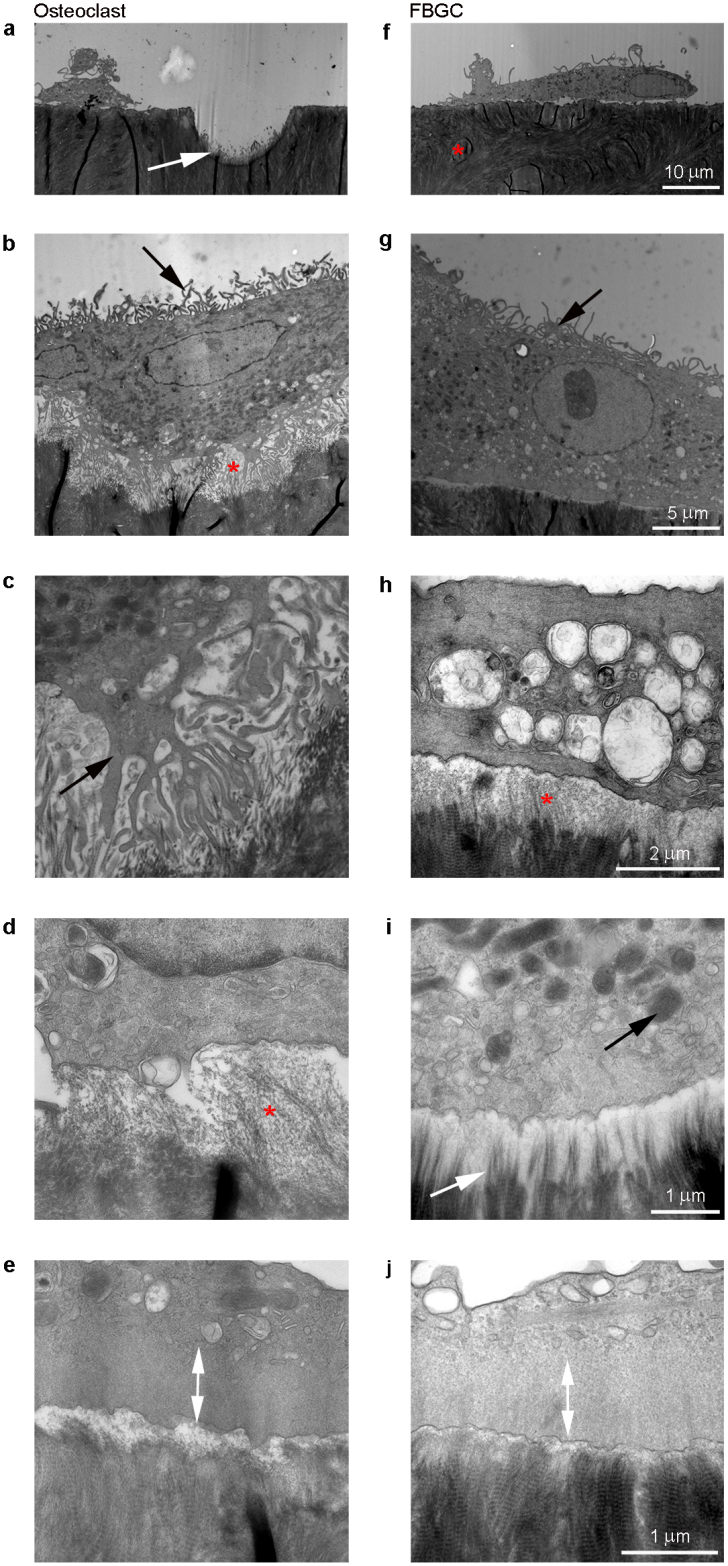
Transmission electron microscopy of osteoclasts and FBGCs. In the osteoclast cultures, resorption pits were visible with exposed collagen fibrils protruding from the pit surface (**a**; white arrow). No resorption pits were seen in the FBGC cultures (**f;** red asterisk = bone). The osteoclasts showed an extensive ruffled border with on both sides a sealing zone (**b**; red asterisk, **c**; black arrow). No ruffled border was seen in the FBGCs (**f, g**). On the basolateral side of the membrane, both cell types showed “finger”-like structures (**b, g**; black arrow). For FBGCs, these membrane protrusions were generally longer and more abundant than those of osteoclasts. Adjacent to the FBGCs, an electron translucent area was seen covering the bone surface (**h**; red asterisk), suggesting demineralization. In this area, collagen fibils were exposed (**i**; white arrow). Both cell types contained high numbers of mitochondria (**b, c, g, i**; black) and the presence of a sealing zone approximatley 1 μm wide where no organelles were present (**e, j**; white arrow). Scale bar is 10 μm for panels **a** and **f**; 5 μm for panels **b** and **g**; 2 μm for panels **c** and **h**; and 1 μm for panels **d**, **e**, **i** and **j**.

Osteoclasts and FBGCs generated on bone slices exhibited a similar ultrastructural morphology. Both cell types were firmly attached to the bone surface and contained numerous electron dense mitochondria. Long plasma membrane protrusions resembling “finger”-like structures were visible on the basolateral surface of the FBGCs (Fig 4b and 4g). No “finger”-like structures were visible on the apical surface, the side facing the bone. These “finger”-like structures were also present on the basolateral surface of osteoclasts (Fig 4b), but they were less frequent and the “fingers” were shorter. Another resemblance in the ultrastructural morphology between the two cells was the presence of condensed areas that lacked organelles; resembling a sealing zone present on the apical side of the cell facing the bone surface (Fig 4e and 4j).

A striking difference between the cell types was the presence of a ruffled border, which was only apparent in resorbing osteoclasts (Fig 4b and 4c). Bone resorption was clearly occurring adjacent to the ruffled border of the osteoclasts, evidenced by the generation of Howship’s lacunae and clear signs of degradation of the bone (Fig 4a, 4b and 4c). The lacunae were mostly steeply concave and approximately 10 μm deep. In contrast, such lacunae were never observed in the FBGC cultures (Fig 4f). However, at the interface of FBGCs and bone, a thin area of the bone surface (approximately 500 nm deep) was less electron dense than adjacent stretches of bone not populated by FBGCs, indicating a lack of bone mineral (Fig 4h and 4i). The collagen of the bone was still intact (Fig 4i). This electron-sparse band appeared similar to the bottom of the Howship’s lacunae formed by the osteoclasts. Such a mineral devoid area was only seen adjacent to the osteoclasts and FBGCs and not on control bone slices.

### Gene expression revealed genes necessary for acidification

**Fig 5:**
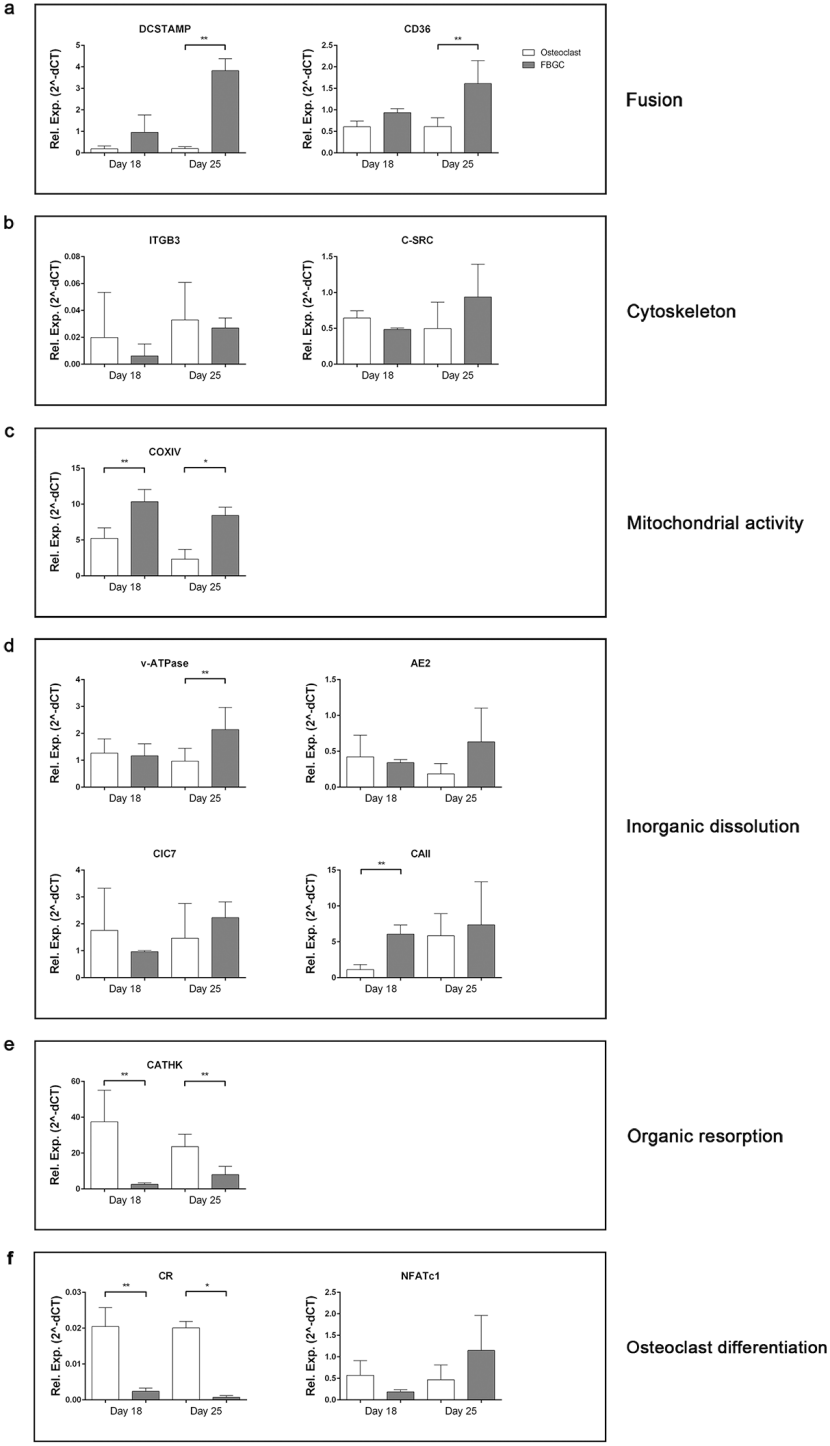
Osteoclast and FBGC mRNA expression at different time points. Data represent the mean ± S.D. of n = 5 donors. *p<0.05, **p<0.01.

We analyzed the expression of genes related to cell-cell fusion, adhesion, mitochondrial activity, acidification and osteoclast differentiation and function at 18 and 25 days of the culture (Fig 5). DC-STAMP, necessary for fusion of osteoclasts and FBGCs [16] and CD36 (fusion of FBGC) [27], was expressed at both time points (day 18 and day 25). At day 18 no difference was observed between the osteoclasts and the FBGCs, however, at day 25 DC-STAMP and CD36 were significantly upregulated in the FBGCs compared to the osteoclasts (Fig 5a; p<0.01). Beta-3 integrin (attachment), and proto-oncogene tyrosine-protein kinase Src (c-Src) (cytoskeleton reorganization, podosomes) [28] were both expressed but there was no difference between the two cell types (Fig 5b).

Cytochrome c oxidase (COXIV), an enzyme for mitochondrial activity, was significantly higher in the FBGC cultures at both time points (Fig 5c; p<0.05 at day 18 and p<0.01 at day 25) compared to the osteoclast.

Two important genes for lowering the pH, CAII and v-ATPase were both upregulated in the FBGC compared to the osteoclast. CAII at day 18 (p<0.01) and v-ATPase at day 25 (p<0.01) (Fig 5d).

Cathepsin K and calcitonin receptor (CR), both genes relatively specific for osteoclasts, were significantly upregulated in osteoclasts compared to the FBGC. Cathepsin K at day 18 and day 25, p<0.01 and CR at day 18, p<0.01 and day 25, p<0.05 (Fig 5f). In the FBGC these genes were hardly expressed.

Nuclear factor of activated T-cells, cytoplasmic 1 (NFATc1), a transcription factor for osteoclast differentiation, was expressed comparably in both cell types (Fig 5f).

### FBGCs dissolved parts of a biomimetic hydroxyapatite coating

The above findings on the expression of genes (Fig 5d) necessary for acidification as well as the TEM observations (Fig 4h and 4i) strongly suggest that the FBGCs possess the machinery to dissolve mineral.

**Fig 6:**
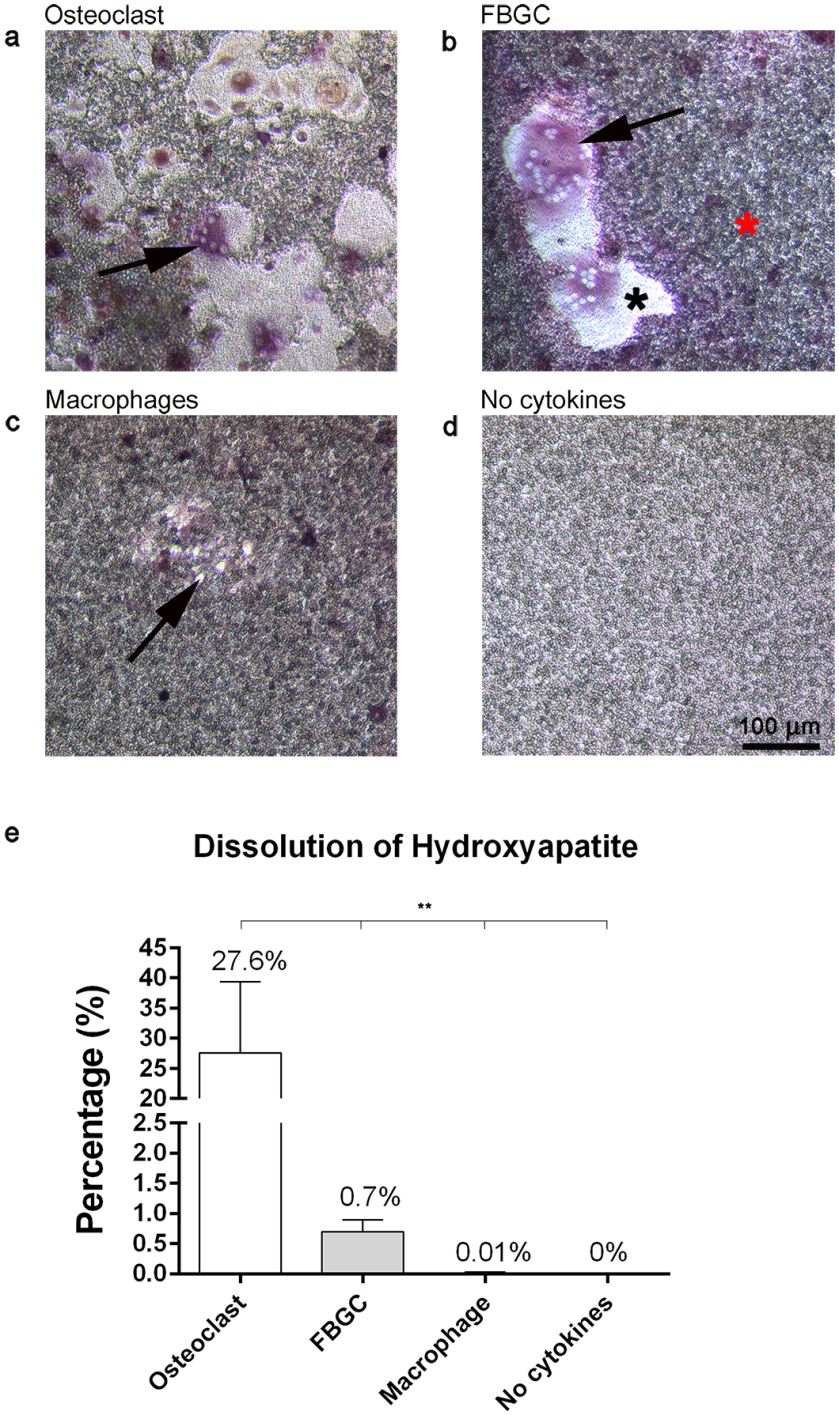
Osteoclasts and FBGCs cultured on biomimetic hydroxyapatite coatings. After 25 days of culture, cells were stained for TRAcP activity and nuclei (DAPI). Multinucleated, TRAcP positive osteoclasts (**a**; black arrow) and FBGCs (**b**; black arrow) dissolved the coating (coating; red asterisk, plastic; black aterisk). Macrophages were also able to dissolve small parts of the coating (**c**; black arrow, **e**). Control wells, incubated without cells, showed no signs of apatite coating dissolution (**d**). Quantitatively, osteoclasts dissolved more of the coating than the FBGCs (**e**). Percent dissolution of the hydroxyapatite coating plots represent the mean ± S.D. per 0.32 cm^2^ coating surface. Scale bar = 100 μm. *p<0.05, **p<0.01.

To further test such a capacity we used plates with a calcium phosphate coating which mimics the hydroxyapatite in bone [22]. The osteoclasts proved to dissolve the coating extensively (Fig 6a). Almost 28% (Fig 6e) was dissolved. Multiple osteoclasts were observed in the dissolved area (i.e. on plastic). The dissolved areas were of different sizes and randomly spread throughout the well. Remarkably, the FBGCs dissolved the coating also (Fig 6b), multiple FBGCs were seen in these areas, but the total dissolved area proved to be far less than with the osteoclasts (27.6% ± 11.7% SD vs 0.7% ± 0.17% SD) (Fig 6e). These dissolved areas were more regularly shaped compared to the dissolved areas induced by osteoclasts. Macrophages dissolved only very small spots of the coating (0.01% ± 0.01% SD) (Fig 6c and 6e). The control cultures (no cytokines added, fibroblasts (not shown) and only medium) did not reveal any dissolution of the coating (Fig 6d and 6e).

### Blocking of v-ATPase activity prevented dissolution of the mineral coating

**Fig 7:**
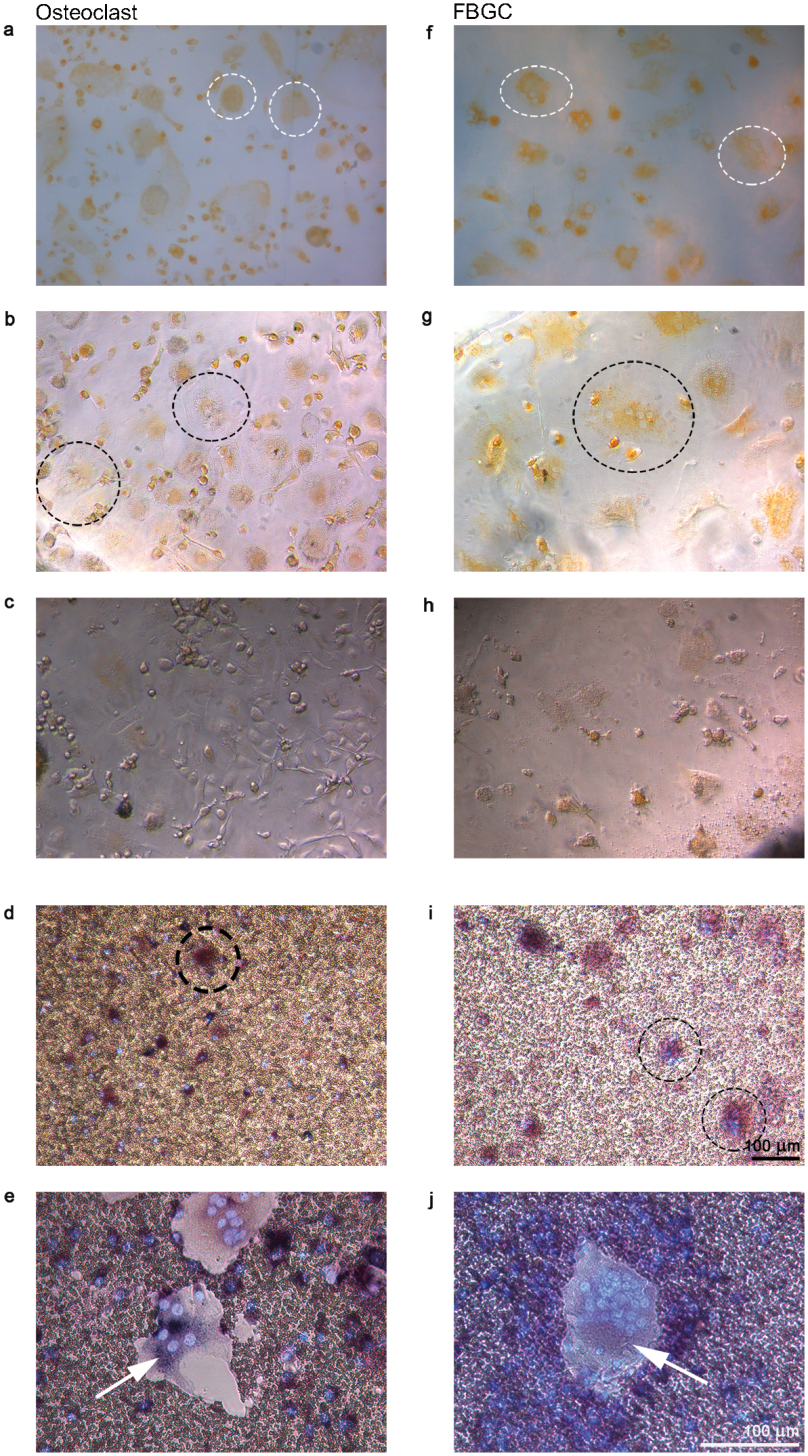
Resorption activity of osteoclasts and FBGCs on biomimetic hydroxyapatite coatings after concanamycin A treatment. Cells were cultured for 25 days and stained with acridine orange to visualize sites with low pH. Osteoclasts (left column) and FBGCs (right column) stained positive for acridine orange on both bone (**a, f**; white dashed circles) and tissue culture plastic (**b, g**; black dashed circles). After incubation with concanamycin A, acridine orange-positive vacuoles were hardly detected (**c, h**); moreover, dissolution of hydroxyapatite was blocked (**d, I**; osteoclasts and FBGCs are visible in black dashed circles) compared to control cells cultured without concamycin A (**e, j**). Cells were stained for TRAcP and DAPI. Scale bar = 100 μm.

Acidic lysosomal compartments were visualized with acridine orange in both cell types on bone (Fig 7a and 7f) and on plastic (Fig 7b and 7g). Blocking of v-ATPase with ConcA resulted in an almost complete abrogation of acridine orange staining (Fig 7c and 7h). We analyzed this on plastic because it was not possible to visualize the cells on bone due to the lack of staining of acridine orange in the presence of ConcA.

Culturing the cells on the biomimetic hydroxyapatite coating and subsequently blocking of v-ATPse inhibited the dissolution of the coating in both cell cultures (Fig 7d and 7i). Without the inhibitor the osteoclasts and FBGCs did dissolve the coating (Fig 7e and 7f). The cells incubated with ConcA did proliferate and survived (Fig 7c, 7d, 7h and 7i).

## Discussion

To our knowledge, this is the first report in which the function of osteoclasts and FBGCs, two closely related multinucleated cell types, was directly analyzed and compared in vitro on bone. Both cell types were generated on bone slices by differentiating their common precursor, the CD14^+^ monocyte, using different sets of cytokines. These cell types shared similarities in terms of TRAcP activity, multinuclearity, morphology, cytoskeletal organization, and gene expression. Yet, an important difference between the two cell types was the inability of FBGCs to resorb bone. These cells could, however, dissolve the mineral fraction of bone as was shown on bone slices and by using a biomimetic hydroxyapatite coating.

An important similarity between the two cell types was the high expression of TRAcP activity. TRAcP is a classical osteoclast marker implicated with bone resorption but it may have other functions such as catalyzing reactive oxygen species (ROS) production [29]. ROS has been shown to be a marker of activated macrophages and serves to break down foreign bodies [30–32], so in this way TRAcP and ROS may function uniquely in osteoclasts and FBGCs to resorb or dissolve bone or foreign bodies, respectively. Whether TRAcP functions differently between these two cell types cannot be determined with our results; however, because the FBGC could not resorb bone despite high TRAcP activity, it may be that the enzyme is primarily involved in ROS production. Further studies are needed to explore this possibility.

In addition to TRAcP, another distinctive feature of both cell types is their multinuclearity and their large size. FBGCs were, however, much larger and contained more nuclei per cell than osteoclasts. This difference in size may be explained by the difference in expression of fusion proteins—in particular, FBGCs expressed significantly higher levels of DC-STAMP and CD36 than osteoclasts. It is demonstrated that, in osteoclasts and FBGCs, inhibition of DC-STAMP results in complete abrogation of cell-cell fusion [4,13,16], whereas CD36 impairs macrophage fusion [27]. Therefore, it seems plausible that the higher expression of these proteins in FBGCs resulted in increased cell-cell fusion.

Another similarity between the two cell types was the presence of actin rings, podosome belts and sealing zones. This finding was remarkable for the FBGCs because these structures are considered to be specific for osteoclasts due to their apparent role in bone resorption. It has been shown that resorption depends on the organization of F-actin structures (actin rings) into a sealing zone and that this structure is required for the formation of a resorption area [33,34]. Our findings appear to suggest that both cell types have a similar capacity to resorb bone. However, despite their formation of actin rings, sealing zones, multinuclearity, and TRAcP activity, FBGCs were unable to resorb bone like osteoclasts. Notable differences between these cell types were the lack of a ruffled border and low cathepsin K expression by FBGCs, both of which could explain why the latter cell type did not resorb the bone. If an osteoclast lacks a ruffled border and/or cathepsin K, bone resorption does also not occur, a condition known as osteopetrosis [35,36].

Surprisingly, although FBGC did not resorb the bone, they were able to demineralize superficial areas of the bone surface. In this area the bone matrix was still present but mineral appeared to be dissolved, evidenced in TEM by shallow stretches of translucent bone substrate. This capacity to demineralize bone was confirmed by their ability to dissolve discreet areas of an apatite coating designed to mimic bone mineral. These results coupled with their high expression of proteins involved in the acidifying machinery such as v-ATPase, CIC-7, AE2, and CAII, suggest that FBGCs could acidify a mineralized substrate in a similar way as the osteoclast. In fact, FBGC expression of these genes was even higher than that in the osteoclast.

Similar to osteoclasts [24,37,38], FBGCs contained numerous acidic vacuoles in their cytosol as shown by acridine orange staining, indicating their ability to create acidified compartments. In osteoclasts, the acidification of the resorption compartment is mainly driven by H^+^ transport by membrane-bound v-ATPase proton pumps. To probe the mechanism by which FBGCs can resorb bone mineral, v-ATPase blockade with ConcA stunted hydroxyapatite resorption by FBGCs and osteoclasts, indicating that FBGCs like osteoclasts dissolve mineralized substrates in a v-ATPase dependent manner likely through acidification.

Proton transport by v-ATPase incurs a high metabolic load, supplied by ATP in the mitochondria [39,40]. In line with this, ATP production by osteoclasts is correlated with bone resorption [41]. Here, we observed high numbers of mitochondria present in both FBGCs and osteoclasts, along with enhanced expression of the mitochondrial marker COXIV. Together, these results further substantiate the proposed mechanism that FBGCs, like osteoclasts, acidify compartments opposed to their basal membranes with the necessary acidification machinery to dissolve bone mineral.

The FBGCs were, however, not as efficient as the osteoclasts in dissolving the coating; far more coating was dissolved by the osteoclasts. A possible explanation for this difference could be that FBGCs did not form a ruffled border. Proton pumps of osteoclasts are concentrated in the membrane of the ruffled border, thus making it possible for this cell type to efficiently dissolve mineral at that site. Since FBGCs did not form a ruffled border their proton pumps are probably less concentrated in certain areas of the membrane of the FBGCs. Therefore, the osteoclast is better suited for its task to dissolve inorganic substrates. Prior work from our group has also shown how FBGCs may be differently affected by surface topography than osteoclasts, which could also explain differences in their resorption [7]. To explain these differences and to understand the interactions of FBGCs with biomaterials more studies should be done.

## Conclusion

The findings presented here indicate a previously unknown capacity of FBGCs, which was thought to be exclusive to osteoclasts: the ability to dissolve bone mineral. This process appears to depend, as with the osteoclast, on the activity of v-ATPase. However, FBGCs cannot degrade the organic part of bone. Similar to osteoclasts, FBGCs can reorganize their cytoskeleton to orient with their substrate by forming actin rings. Our results imply that biomaterials composed of calcium phosphates (e.g., bone graft substitutes) or implants coated with calcium phosphates, such as a metallic joint prosthetics coated with HA, may be susceptible to degradation or loosening due to FBGC dissolution of the mineral surface. A
